# Clinical outcomes associated with Shenling Bufei Tongluo Decoction as add-on therapy in stable COPD patients with a frequent-exacerbator phenotype: a retrospective real-world cohort study

**DOI:** 10.3389/fmed.2026.1865770

**Published:** 2026-07-08

**Authors:** Xin Hou, Wenxuan Wang, Lijian Pang

**Affiliations:** 1 First Clinical College, Liaoning University of Traditional Chinese Medicine, Shenyang, China; 2 Department of Intensive Care Rehabilitation, Liaoning University of Traditional Chinese Medicine Affiliated Hospital, Shenyang, China; 3 Department of Critical Care Medicine for Respiratory and Pulmonary Diseases, Liaoning University of Traditional Chinese Medicine Affiliated Hospital, Shenyang, China

**Keywords:** chronic obstructive pulmonary disease, frequent-exacerbator phenotype, propensity score weighting, real-world evidence, Shenling Bufei Tongluo Decoction, traditional Chinese medicine

## Abstract

**Background:**

Acute exacerbations accelerate disease progression and increase mortality in chronic obstructive pulmonary disease (COPD). Patients with a frequent-exacerbator phenotype remain at persistently high risk despite conventional therapies, highlighting the need for effective adjunctive strategies. Shenling Bufei Tongluo Decoction (SLBTD) is used in routine clinical practice, but real-world evidence regarding its clinical outcomes in this high-risk population remains limited.

**Methods:**

This retrospective real-world cohort study evaluated SLBTD add-on therapy in stable COPD patients with a frequent-exacerbator phenotype. Patients receiving SLBTD plus conventional management were compared with those receiving conventional management alone. Propensity score weighting was applied to balance baseline covariates. The primary outcome was first moderate-to-severe COPD exacerbation during a 1-year post-index observation period. Secondary outcomes included annualized exacerbation rates, hospitalization, symptoms, pulmonary function, traditional Chinese medicine (TCM) syndrome score, and recorded safety outcomes.

**Results:**

Among 528 eligible patients (176 SLBTD, 352 comparator), stabilized weighting achieved excellent covariate balance. Patients receiving SLBTD add-on therapy had a lower observed risk of first moderate-to-severe exacerbation [hazard ratio (HR), 0.68; 95% confidence interval (CI), 0.53–0.87; *P* = 0.002], a lower annualized exacerbation rate [rate ratio (RR), 0.71; *P* = 0.001], and fewer severe exacerbations requiring hospitalization [odds ratio (OR), 0.55; *P* = 0.014] than patients receiving conventional management alone. Patients receiving SLBTD also showed more favorable changes in COPD Assessment Test (CAT) score, modified Medical Research Council (mMRC) dyspnea grade, forced expiratory volume in 1 s (FEV_1_)% predicted, and TCM syndrome score (all *P* < 0.01). Sensitivity analyses using 30-day landmark, time-varying exposure, restricted-cohort, and severe-exacerbation-only definitions yielded consistent results. Recorded safety outcomes were comparable between groups.

**Conclusion:**

In routine clinical practice, patients receiving SLBTD add-on therapy had a lower recorded exacerbation burden and more favorable symptom-related outcomes than those receiving conventional management alone, without an observed increase in recorded safety events. These findings should be interpreted as observational associations rather than evidence of causal efficacy.

## 1 Introduction

Chronic obstructive pulmonary disease (COPD) is a heterogeneous and progressive respiratory disease characterized by persistent respiratory symptoms, airflow limitation, and recurrent clinical deterioration. Acute exacerbations are pivotal events in the natural history of COPD because they accelerate lung function decline, increase symptom burden, drive emergency department visits and hospitalizations, and contribute to subsequent mortality and readmission risk (1–3). Recent real-world evidence also shows that COPD exacerbations remain a major source of inpatient healthcare utilization, with substantial variation in investigation and treatment practices across clinical settings (4). Reducing exacerbation burden is therefore a central objective of stable-phase COPD management. A clinically important subgroup of patients has a frequent-exacerbator phenotype, defined by recurrent exacerbations despite being outside an acute episode. This phenotype is clinically relevant because previous exacerbation history is one of the strongest predictors of future exacerbation risk, and patients with recurrent exacerbations have persistently higher risk of subsequent adverse outcomes (5, 6). Recent studies have further emphasized the need for risk stratification using routinely available clinical data in patients at risk for acute exacerbations (7). Stable COPD patients with a frequent-exacerbator phenotype therefore represent a high-risk population in whom add-on management strategies require careful real-world evaluation.

Chinese herbal medicine is commonly used as add-on therapy in routine COPD care in China. Previous randomized studies of Chinese herbal formulas, including YuPingFeng granules, JianPiYiFei II granules, Bu-Fei Yi-Shen granules, and traditional Chinese medicine (TCM) combined with conventional Western medicine, have reported potential benefits for exacerbation reduction, symptom improvement, quality of life, or hospitalization-related outcomes in selected COPD populations (8–11). However, evidence remains formula-specific and population-specific. In particular, real-world evidence for Shenling Bufei Tongluo Decoction (SLBTD) among stable COPD patients with a frequent-exacerbator phenotype is limited. Given the inflammatory and immune dysregulation underlying COPD heterogeneity and exacerbation susceptibility, further evaluation of add-on therapies in clinically defined high-risk populations is warranted (12). Therefore, this study aimed to evaluate clinical outcomes associated with SLBTD add-on therapy among stable COPD patients with a frequent-exacerbator phenotype in routine clinical practice. We conducted a single-center retrospective real-world cohort study using electronic medical records, prescription records, pulmonary function reports, laboratory data, and follow-up records. The primary outcome was first moderate-to-severe COPD exacerbation during the 365-day post-index observation period. Secondary outcomes included exacerbation rate, hospitalization, emergency department visits, COPD-related medication use, symptom scores, pulmonary function indices, TCM syndrome score, and recorded safety outcomes. Propensity score weighting was used to reduce measured baseline imbalance between patients receiving SLBTD add-on therapy and those receiving conventional COPD management without SLBTD.

## 2 Materials and methods

### 2.1 Study design, setting, and ethics

This single-center retrospective real-world cohort study was conducted at the Affiliated Hospital of Liaoning University of Traditional Chinese Medicine using existing clinical records from routine care. The study followed the Strengthening the Reporting of Observational Studies in Epidemiology (STROBE) guideline for cohort studies (13). Data were extracted from 1 January 2019 to 31 December 2025. Eligible index dates were restricted to 1 January 2020 through 31 December 2024, allowing a 365-day baseline assessment window and a 365-day post-index outcome ascertainment window for each patient. Treatment was not assigned by the investigators, prescription duration was not determined by study protocol, and no study-mandated follow-up was implemented. Exposure was defined retrospectively from routine electronic prescription records; therefore, this study was not registered as a clinical trial. The protocol was approved by the Ethics Committee of the Affiliated Hospital of Liaoning University of Traditional Chinese Medicine [Approval No. 2025061FS(KT)-030-02]. De-identified retrospective data were analyzed, and written informed consent was waived.

### 2.2 Data source and cohort identification

Data were extracted from the hospital electronic medical record system, outpatient records, inpatient records, electronic prescription records, pulmonary function reports, laboratory information systems, and structured follow-up records. Patient-level records were de-duplicated using the unique hospital medical record identifier. For patients with multiple eligible visits, only one index date was assigned according to the exposure and comparator definitions described below. Patients were eligible for inclusion if they met all of the following criteria: (1) age 40 years or older at the index date; (2) physician-diagnosed chronic obstructive pulmonary disease (COPD); (3) spirometric confirmation of COPD, defined as post-bronchodilator forced expiratory volume in 1 s (FEV_1_) to forced vital capacity (FVC) ratio <0.70 (14); (4) stable COPD at the index visit; (5) frequent-exacerbator phenotype based on exacerbation history during the 365 days before the index date; and (6) available clinical records for baseline covariate extraction and post-index outcome ascertainment. Patients were excluded if they had an acute exacerbation at the index date, asthma as the primary respiratory diagnosis, lung cancer, active pulmonary tuberculosis, interstitial lung disease, missing exposure status, missing index date, missing primary outcome information, or concurrent use of other COPD-targeted oral Chinese herbal formulas that prevented clear exposure classification.

### 2.3 Definitions of stable COPD and frequent-exacerbator phenotype

Stable COPD was defined as COPD without worsening respiratory symptoms requiring systemic corticosteroids, antibiotics, emergency department attendance, or hospitalization within 4 weeks before the index date. A moderate acute exacerbation was defined as worsening respiratory symptoms requiring treatment with systemic corticosteroids and/or antibiotics without hospitalization. A severe acute exacerbation was defined as an exacerbation requiring emergency department attendance or hospitalization. These definitions followed contemporary Global Initiative for Chronic Obstructive Lung Disease (GOLD) criteria for COPD diagnosis and exacerbation classification (14). The frequent-exacerbator phenotype was defined as two or more moderate acute exacerbations or at least one severe acute exacerbation during the 365 days before the index date. This definition was selected to identify a clinically high-risk and relatively homogeneous population with recurrent or severe exacerbation history and is consistent with the established COPD frequent-exacerbator phenotype, which has traditionally been defined using a threshold of two or more moderate exacerbations or at least one severe exacerbation in the previous year (5, 6, 14). We also acknowledge that the latest GOLD 2026 report has updated the A/B/E assessment framework by recognizing that even one moderate exacerbation may increase the risk of subsequent events and should prompt consideration of treatment escalation (15). However, because the objective of the present study was to evaluate SLBTD add-on therapy in patients with a stricter frequent-exacerbator phenotype rather than all patients meeting the broader treatment-escalation threshold, the prespecified definition of two or more moderate exacerbations or at least one severe exacerbation was retained.

### 2.4 Exposure, comparator, index date, and observation window

The exposure group consisted of patients who received Shenling Bufei Tongluo Decoction (SLBTD) as add-on therapy to conventional COPD management during routine clinical care. The comparator group consisted of eligible patients who received conventional COPD management without SLBTD at the index visit and without an SLBTD prescription within the first 30 days after the index date. Conventional COPD management included maintenance inhaled bronchodilator therapy, inhaled corticosteroid-containing regimens, mucolytic therapy, long-term oxygen therapy, pulmonary rehabilitation, antibiotics, and systemic corticosteroids when clinically indicated. For patients in the SLBTD group, the index date was the first stable COPD visit during the index period at which SLBTD was prescribed. For comparator patients, the index date was the first eligible stable COPD visit during the same index period without an SLBTD prescription. The 365 days before the index date constituted the baseline assessment window, and the 365 days after the index date constituted the post-index outcome ascertainment window. SLBTD exposure was identified from electronic prescription records. Only prescriptions containing the fixed 12-component SLBTD formula listed in Table 1 were classified as SLBTD exposure. Patients receiving other COPD-targeted oral Chinese herbal formulas or respiratory-targeted Chinese patent medicines were excluded to maintain clear exposure classification. Prescription duration was calculated from electronic prescription dates and recorded days supplied. We additionally reviewed hospital procedure records and clinical charts to identify concomitant non-oral TCM-related interventions during the post-index observation period, including acupoint application, acupuncture, moxibustion, cupping, auricular therapy, and standardized hospital-directed TCM traditional exercise rehabilitation. Oral Chinese patent medicines prescribed exclusively for non-respiratory comorbidities were not classified as COPD-targeted TCM therapy but were reviewed and assessed for between-group balance.

**Table 1:** Composition and prescription characteristics of Shenling Bufei Tongluo Decoction.

Herb name	Latin pharmaceutical name	Medicinal part	Daily dose (g)	Functional role in formula
Renshen	Ginseng Radix et Rhizoma	Root and rhizome	15	Tonifying qi and supporting lung deficiency
Baifuling	Poria	Sclerotium	15	Strengthening the spleen and resolving dampness
Baizhu	Atractylodis Macrocephalae Rhizoma	Rhizome	15	Strengthening the spleen and tonifying qi
Shanyao	Dioscoreae Rhizoma	Rhizome	15	Tonifying the spleen and lung
Lianzirou	Nelumbinis Semen	Seed	9	Strengthening the spleen and supporting deficiency
Yiyiren	Coicis Semen	Seed	9	Draining dampness and strengthening the spleen
Jiegeng	Platycodonis Radix	Root	6	Diffusing lung qi and guiding the formula to the lung
Gancao	Glycyrrhizae Radix et Rhizoma	Root and rhizome	9	Harmonizing the formula
Hongjingtian	Rhodiolae Crenulatae Radix et Rhizoma	Root and rhizome	10	Tonifying qi and supporting lung function
Jixueteng	Spatholobi Caulis	Stem	10	Activating blood and dredging collaterals
Dilong	Pheretima	Dried body	10	Dredging collaterals
Niuxi	Achyranthis Bidentatae Radix	Root	15	Activating blood and guiding downward
**Prescription characteristic**		**Description**		
Dosage form		Traditional Chinese herbal decoction		
Route of administration		Oral administration		
Administration frequency		Twice daily		
Dose basis		The listed amounts represent the daily crude-herb dose recorded in the hospital prescription system		
Prescription source		Electronic prescription records from routine clinical care		
Exposure classification		Prescription of the fixed 12-component Shenling Bufei Tongluo Decoction formula at the eligible stable COPD index visit		
Treatment duration		Calculated from prescription dates and recorded days supplied in the electronic prescription records		
Concomitant Chinese herbal formula rule		Patients receiving other COPD-targeted oral Chinese herbal formulas were excluded to maintain clear exposure classification		

Latin pharmaceutical names were standardized according to Chinese materia medica nomenclature in the Pharmacopeia of the People's Republic of China (28). COPD, chronic obstructive pulmonary disease.

### 2.5 Outcomes

The primary outcome was the first documented moderate-to-severe COPD exacerbation during the 365-day post-index observation period. The event date was defined as the earliest date of an eligible moderate exacerbation encounter, emergency department visit, hospital admission, or COPD-related systemic corticosteroid and/or antibiotic prescription. Secondary event outcomes included the total number of moderate-to-severe exacerbations, severe exacerbation requiring hospitalization, COPD-related hospitalization, all-cause hospitalization, emergency department visit for COPD, systemic corticosteroid use, and antibiotic use during the post-index observation period. Symptom, pulmonary function, and TCM-related outcomes included COPD Assessment Test (CAT) score, modified Medical Research Council (mMRC) dyspnea grade, FEV_1_% predicted, FEV_1_/FVC, and TCM syndrome score. CAT and mMRC were used to assess COPD health status and dyspnea-related functional limitation, respectively (16, 17). For these measures, baseline values were defined as the closest records within 90 days before or on the index date, and post-index values were defined as records closest to day 365 within the day 270 to day 455 window. Patients without paired measurements were excluded only from the corresponding outcome analysis. Safety outcomes included recorded adverse events, gastrointestinal symptoms, allergic reaction, dizziness, palpitations, prescription-recorded treatment discontinuation, all-cause death, and laboratory safety abnormalities during the post-index observation period. Safety outcomes were obtained through retrospective passive monitoring based on outpatient records, inpatient records, laboratory information systems, and prescription records, rather than through active prospective adverse-event surveillance. Laboratory abnormalities were defined as alanine aminotransferase or aspartate aminotransferase greater than three times the upper limit of normal, serum creatinine increase of at least 50% from baseline, or blood urea nitrogen above the upper reference limit.

### 2.6 Baseline covariates and missing data

Baseline covariates were measured before or on the index date and were selected according to clinical relevance, data availability in routine records, and their potential association with SLBTD prescribing and COPD outcomes. Covariates included age, sex, body mass index, smoking status, COPD duration, previous moderate and severe exacerbations, previous COPD-related hospitalization, FEV_1_% predicted, FEV_1_/FVC, GOLD airflow limitation grade, CAT score, mMRC dyspnea grade, TCM syndrome score, baseline COPD management, comorbidities, and laboratory indicators. Baseline COPD management included inhaled corticosteroid (ICS)-containing inhaled therapy, long-acting beta_2_-agonist/long-acting muscarinic antagonist (LABA/LAMA) therapy, triple inhaled therapy, long-term oxygen therapy, pulmonary rehabilitation, systemic corticosteroid use, and antibiotic use during the baseline window. Comorbidities included hypertension, diabetes mellitus, coronary heart disease, chronic heart failure, and chronic kidney disease. Laboratory indicators included white blood cell count, blood eosinophil count, C-reactive protein, serum albumin, and serum creatinine. Calendar year of the index date was also included in the propensity score model to partially account for secular changes in clinical practice, healthcare access, and pandemic-period effects during the study period. For pulmonary function, symptom scores, TCM syndrome score, and laboratory variables, the most recent valid value before or on the index date was used.

Several clinically relevant variables were reviewed but were not included in the primary propensity score model because they were not consistently or systematically recorded in the retrospective electronic medical record system. Smoking intensity, such as pack-years or cigarettes per day, was incompletely documented in structured fields; therefore, smoking status was included, but smoking intensity could not be reliably modeled. Influenza and pneumococcal vaccination histories were not comprehensively captured because many vaccinations were administered outside the hospital or in community settings. Direct socioeconomic indicators, including income, education, occupation, and household support, were not routinely available in the hospital records. Although baseline long-term oxygen therapy was included as a treatment-related covariate, adherence to home oxygen therapy, including daily oxygen-use duration and family supervision or support, was not consistently documented. Coronavirus disease 2019 (COVID-19) infection status was identifiable when documented in hospital records, but infections diagnosed outside the hospital or not resulting in recorded clinical encounters could not be comprehensively ascertained. These variables were therefore not included in the primary propensity score model to avoid relying on highly incomplete and non-systematically collected covariates.

Patients with missing exposure status, index date, or primary outcome information were excluded. Missing baseline covariates were handled using multiple imputation by chained equations (MICE) with 20 imputed datasets (18). The imputation model included exposure status, primary outcome status, time to event or censoring, baseline covariates, and major secondary event outcomes. Multiple imputation was restricted to baseline covariates and was not used to impute exposure status, index dates, or the primary post-index clinical event outcome. Continuous variables were imputed using predictive mean matching, and ordinal variables were imputed using ordinal logistic regression. Variables with more than 30% missingness were not included in the primary propensity score or outcome models. The extent of missingness and the imputation approach for each baseline covariate are summarized in Supplementary Table S1. Patients without paired symptom, pulmonary function, or TCM syndrome measurements were excluded only from the corresponding change-score analyses. To assess the stability of missing-data handling, we performed methodological sensitivity analyses comparing the primary MICE analysis with a complete-case analysis, an expanded MICE analysis using 40 imputed datasets, and an alternative-seed MICE analysis. These analyses were used to evaluate whether the primary association was materially altered by the imputation specification.

### 2.7 Propensity score weighting and covariate balance

Because SLBTD was prescribed during routine clinical care rather than assigned by the investigators, inverse probability of treatment weighting based on the propensity score was used to reduce measured baseline imbalance between exposure groups (19, 20). The propensity score was estimated using multivariable logistic regression with receipt of SLBTD as the dependent variable. The propensity score model included age, sex, body mass index, smoking status, COPD duration, previous moderate exacerbations, previous severe exacerbations, previous COPD-related hospitalization, FEV_1_% predicted, FEV_1_/FVC, GOLD airflow limitation grade, CAT score, mMRC dyspnea grade, inhaled corticosteroid-containing therapy, long-acting bronchodilator therapy, triple inhaled therapy, long-term oxygen therapy, pulmonary rehabilitation, major comorbidities, blood eosinophil count, C-reactive protein, serum albumin, renal function, liver function, baseline systemic corticosteroid use, baseline antibiotic use, and calendar year of the index date.

Stabilized inverse probability of treatment weights were calculated as follows:


$$w_{i}=\{\begin{array}{ll} \frac{\mathrm{Pr}(A=1)}{{\hat{e}}_{i}}, & A_{i}=1,\\ \frac{\mathrm{Pr}(A=0)}{1-{\hat{e}}_{i}}, & A_{i}=0, \end{array}$$

where *A*_*i*_ denotes SLBTD exposure status and ê_*i*_ denotes the estimated propensity score for patient *i*. To limit the influence of extreme weights, stabilized weights were truncated at the 1st and 99th percentiles within each exposure group (20, 21). We examined the distribution of stabilized weights, the sum of stabilized weights, and the effective sample size before and after truncation to assess whether the weighted pseudo-population was dominated by extreme observations or systematically shifted from the original cohort. The effective sample size was calculated as ${\left(\sum w_{i}\right)}^{2}/\sum w_{i}^{2}$. To further evaluate the influence of extreme weights, we compared the primary 1st/99th percentile truncation analysis with analyses using untruncated stabilized weights and stricter 5th/95th percentile truncation.

Covariate balance before and after weighting was assessed using standardized mean differences (SMDs). An absolute SMD below 0.10 was considered acceptable balance. Covariate balance was displayed using a Love plot.

### 2.8 Statistical analysis

No formal sample size calculation was performed because this retrospective study included all eligible patients within the predefined index period. Continuous variables were summarized as mean and standard deviation or median and interquartile range, and categorical variables as frequencies and percentages. Baseline imbalance between exposure groups was assessed using standardized mean differences. Kaplan–Meier curves were used to describe time to first moderate-to-severe COPD exacerbation during the 365-day post-index observation period. The primary association was estimated using a propensity score-weighted Cox proportional hazards model with robust sandwich standard errors. Patients were censored at first moderate-to-severe exacerbation, death, last available clinical record, or day 365. The proportional hazards assumption was evaluated using Schoenfeld residuals, and results were reported as hazard ratios (HRs) with 95% confidence intervals (CIs). Moderate-to-severe exacerbation counts were analyzed using weighted negative binomial regression with observed person-time as an offset and reported as rate ratios (RRs). Binary outcomes, including severe exacerbation requiring hospitalization, COPD-related hospitalization, all-cause hospitalization, emergency department visit for COPD, systemic corticosteroid use, antibiotic use, and recorded safety outcomes, were analyzed using weighted logistic regression and reported as odds ratios (ORs). Changes in CAT score, mMRC dyspnea grade, pulmonary function indices, and TCM syndrome score were analyzed using baseline-adjusted weighted regression models and reported as adjusted mean differences. Four sensitivity analyses were performed for the primary outcome. A 30-day landmark analysis excluded patients with moderate-to-severe COPD exacerbation, death, or censoring within 30 days after the index date, defined exposure during days 0–30, and started follow-up on day 31. A time-varying exposure analysis classified person-time before the first recorded SLBTD prescription as unexposed and person-time thereafter as exposed. To further address potential exposure misclassification caused by treatment cross-over during follow-up, we performed an additional restricted-cohort sensitivity analysis excluding comparator patients who initiated SLBTD therapy between day 31 and day 365. In this restricted analysis, the propensity score model and stabilized inverse probability weights were re-estimated after excluding these patients, and the weighted Cox proportional hazards model for the primary outcome was refitted. A severe-exacerbation-only analysis counted only exacerbations requiring emergency department attendance or hospitalization. These sensitivity analyses were designed to assess whether the primary findings were robust to potential time-related bias, exposure misclassification arising from fixed baseline exposure classification, and the 30-day post-index comparator definition. Prespecified subgroup analyses were conducted by age (<65 vs. ≥65 years), sex, GOLD airflow limitation grade (I–II vs. III–IV), history of severe exacerbation, blood eosinophil count (<300 vs. ≥300 cells/μL), and ICS-containing inhaled therapy. Subgroup analyses used weighted Cox models with treatment-by-subgroup interaction terms. All tests were two-sided, with *P* < 0.05 considered statistically significant. Subgroup analyses were exploratory. Analyses were performed using R version 4.4.2 (R Foundation for Statistical Computing, Vienna, Austria).

## 3 Results

### 3.1 Cohort identification and exposure classification

**Figure 1:**
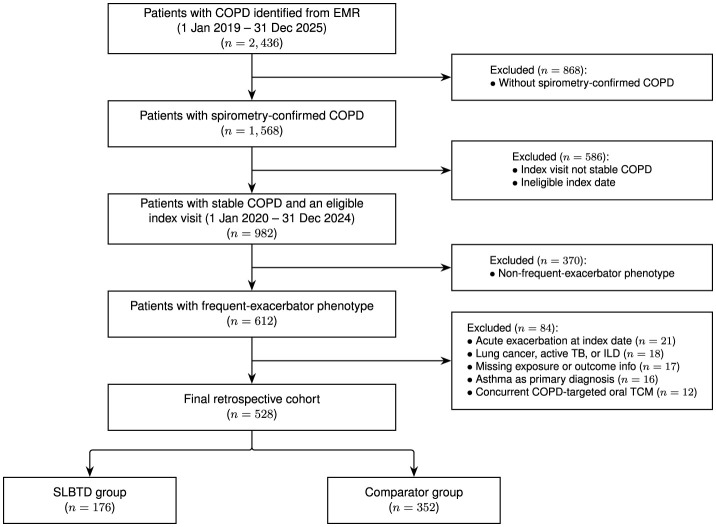
Flow diagram of retrospective cohort identification and exposure classification. COPD, chronic obstructive pulmonary disease; SLBTD, Shenling Bufei Tongluo Decoction.

A total of 2,436 patients with COPD were identified from the electronic medical record system between 1 January 2019 and 31 December 2025. Among them, 1,568 patients (64.4%) had spirometry-confirmed COPD. After restricting the cohort to patients with stable COPD and an eligible index visit between 1 January 2020 and 31 December 2024, 982 patients remained eligible for phenotype assessment. Of these, 612 patients met the definition of a frequent-exacerbator phenotype. A further 84 patients were excluded: 21 had an acute exacerbation at the index date, 16 had asthma as the primary respiratory diagnosis, 18 had lung cancer, active pulmonary tuberculosis, or interstitial lung disease, 17 had missing exposure or primary outcome information, and 12 had unclear exposure classification due to concurrent COPD-targeted oral Chinese herbal formulas. The final retrospective cohort included 528 patients with stable COPD and a frequent-exacerbator phenotype. According to electronic prescription records, 176 patients (33.3%) received Shenling Bufei Tongluo Decoction (SLBTD) as add-on therapy to conventional COPD management, and 352 patients (66.7%) received conventional COPD management without SLBTD. The cohort identification process and exposure classification are shown in Figure 1.

### 3.2 SLBTD treatment duration and concomitant TCM interventions

SLBTD treatment duration and concomitant TCM-related interventions are summarized in Table 2. Among patients receiving SLBTD, the median recorded treatment duration was 112 days [interquartile range (IQR), 64–168]. The treatment-course distribution was as follows: 23 patients (13.1%) received SLBTD for less than 30 days, 31 patients (17.6%) for 30–59 days, 42 patients (23.9%) for 60–89 days, and 80 patients (45.5%) for 90 days or longer. Concomitant non-oral TCM interventions were similarly distributed between the two groups. Any non-oral TCM intervention was recorded in 45 patients (25.6%) in the SLBTD group and 86 patients (24.4%) in the comparator group, with a weighted standardized mean difference of 0.008. Other COPD-targeted oral TCM formulas were not used in either group because such patients were excluded by design. Non-COPD-targeted Chinese patent medicines prescribed for non-respiratory comorbidities were also balanced between the two groups.

**Table 2:** SLBTD treatment duration and concomitant traditional Chinese medicine interventions.

Characteristic	SLBTD group (*n* = 176)	Comparator group (*n* = 352)	Unweighted SMD	Weighted SMD
SLBTD treatment duration				
Median duration, days (IQR)	112 (64–168)	–	–	–
Treatment course distribution, *n* (%)				
<30 days	23 (13.1)	–	–	–
30–59 days	31 (17.6)	–	–	–
60–89 days	42 (23.9)	–	–	–
≥90 days	80 (45.5)	–	–	–
Concomitant non-oral TCM interventions, *n* (%)				
Acupoint application	26 (14.8)	48 (13.6)	0.034	0.011
Acupuncture	11 (6.3)	20 (5.7)	0.024	0.006
Moxibustion	14 (8.0)	25 (7.1)	0.033	0.009
Cupping	8 (4.5)	18 (5.1)	0.027	0.012
Other non-oral TCM^a^	9 (5.1)	15 (4.3)	0.040	0.015
Any non-oral TCM intervention	45 (25.6)	86 (24.4)	0.027	0.008
Other oral TCM therapies, *n* (%)				
Other COPD-targeted oral TCM formulas^b^	0 (0.0)	0 (0.0)	–	–
Non-COPD-targeted Chinese patent medicines^c^	34 (19.3)	71 (20.2)	0.021	0.005

^a^Includes auricular therapy and standardized hospital-directed TCM traditional exercise rehabilitation, such as Baduanjin, recorded in clinical charts.

^b^Patients taking other oral Chinese herbal formulas or patent medicines targeted for respiratory conditions were excluded by design.

^c^Refers to oral Chinese patent medicines prescribed exclusively for non-respiratory comorbidities, such as insomnia, constipation, or osteoarthritis, that were reviewed and assessed for between-group balance.

SLBTD, Shenling Bufei Tongluo Decoction; IQR, interquartile range; SMD, standardized mean difference; TCM, traditional Chinese medicine; COPD, chronic obstructive pulmonary disease.

### 3.3 Baseline characteristics and covariate balance

**Figure 2:**
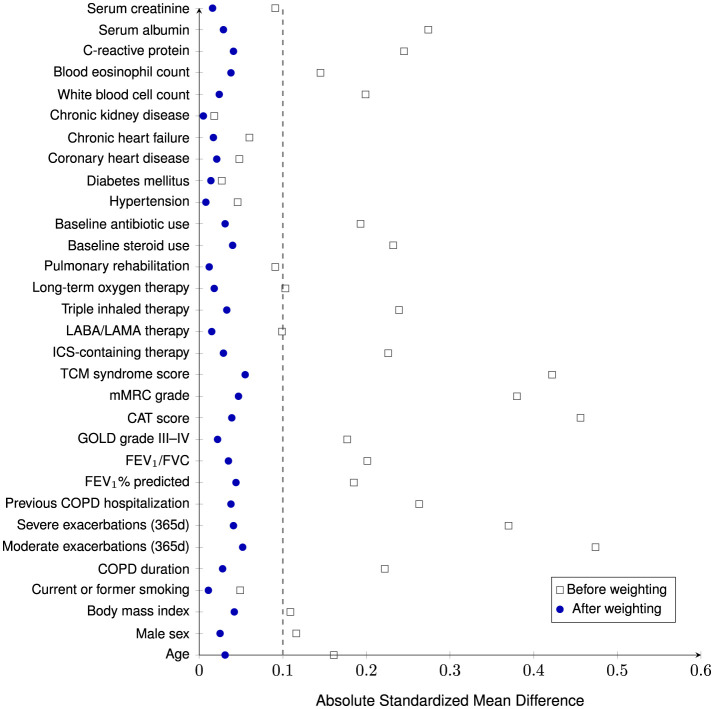
Covariate balance plot before and after propensity score weighting. Standardized mean differences are shown for prespecified baseline covariates. The vertical reference line indicates an absolute standardized mean difference of 0.10. SMD, standardized mean difference.

Baseline characteristics according to exposure status are shown in Table 3. Before propensity score weighting, patients in the SLBTD group had a greater baseline COPD burden than comparator patients. The SLBTD group had longer COPD duration, more moderate and severe exacerbations during the previous 365 days, a higher proportion of previous COPD-related hospitalization, lower pulmonary function, higher CAT score, higher mMRC dyspnea grade, and higher TCM syndrome score. Patients receiving SLBTD were also more likely to receive ICS-containing inhaled therapy, triple inhaled therapy, systemic corticosteroids, and antibiotics during the baseline window. The largest unweighted standardized mean differences were observed for moderate exacerbations in the previous 365 days (SMD = 0.474), CAT score (SMD = 0.456), TCM syndrome score (SMD = 0.422), mMRC dyspnea grade (SMD = 0.380), severe exacerbations in the previous 365 days (SMD = 0.370), serum albumin (SMD = 0.274), and previous COPD-related hospitalization (SMD = 0.263). After stabilized inverse probability of treatment weighting, covariate balance improved substantially. The maximum weighted SMD was 0.055, and all weighted SMDs were below the prespecified threshold of 0.10. Covariate balance before and after weighting is shown in Figure 2.

**Table 3:** Baseline characteristics according to exposure status.

Characteristic	SLBTD group (*n* = 176)	Comparator group (*n* = 352)	Unweighted SMD	Weighted SMD
Age, years	68.4 ± 8.2	67.1 ± 7.9	0.161	0.031
Male sex, *n* (%)	125 (71.0)	268 (76.1)	0.116	0.025
Body mass index, kg/m^2^	23.4 ± 3.8	23.8 ± 3.5	0.109	0.042
Current or former smoking, *n* (%)	136 (77.3)	279 (79.3)	0.049	0.011
COPD duration, years	8.5 ± 4.2	7.6 ± 3.9	0.222	0.028
Moderate exacerbations in previous 365 days	2.6 ± 1.1	2.1 ± 1.0	0.474	0.052
Severe exacerbations in previous 365 days	0.8 ± 0.9	0.5 ± 0.7	0.370	0.041
Previous COPD-related hospitalization, *n* (%)	74 (42.0)	104 (29.5)	0.263	0.038
FEV_1_% predicted	48.6 ± 14.3	51.2 ± 13.8	0.185	0.044
FEV_1_/FVC	53.4 ± 8.7	55.1 ± 8.2	0.201	0.035
GOLD airflow limitation grade III–IV, *n* (%)	101 (57.4)	171 (48.6)	0.177	0.022
CAT score	18.7 ± 5.4	16.3 ± 5.1	0.456	0.039
mMRC dyspnea grade^†^	3 (2–3)	2 (2–3)	0.380	0.047
TCM syndrome score	22.1 ± 5.5	19.8 ± 5.4	0.422	0.055
ICS-containing inhaled therapy, *n* (%)	121 (68.8)	204 (58.0)	0.226	0.029
LABA/LAMA therapy, *n* (%)	68 (38.6)	153 (43.5)	0.099	0.015
Triple inhaled therapy, *n* (%)	83 (47.2)	125 (35.5)	0.239	0.033
Long-term oxygen therapy, *n* (%)	36 (20.5)	58 (16.5)	0.103	0.018
Pulmonary rehabilitation, *n* (%)	21 (11.9)	32 (9.1)	0.091	0.012
Systemic corticosteroid use in previous 365 days, *n* (%)	81 (46.0)	122 (34.7)	0.232	0.040
Antibiotic use in previous 365 days, n (%)	114 (64.8)	195 (55.4)	0.193	0.031
Hypertension, *n* (%)	85 (48.3)	162 (46.0)	0.046	0.008
Diabetes mellitus, *n* (%)	38 (21.6)	72 (20.5)	0.027	0.014
Coronary heart disease, *n* (%)	41 (23.3)	75 (21.3)	0.048	0.021
Chronic heart failure, *n* (%)	24 (13.6)	41 (11.6)	0.060	0.017
Chronic kidney disease, *n* (%)	15 (8.5)	28 (8.0)	0.018	0.005
White blood cell count, × 10^9^/L	7.2 ± 2.1	6.8 ± 1.9	0.199	0.024
Blood eosinophil count, cells/μL^†^	210 (130–310)	185 (115–280)	0.145	0.038
C-reactive protein, mg/L^†^	4.8 (2.1–9.5)	3.5 (1.6–7.2)	0.245	0.041
Serum albumin, g/L	38.4 ± 4.2	39.5 ± 3.8	0.274	0.029
Serum creatinine, μmol/L	82.5 ± 18.4	80.9 ± 16.7	0.091	0.016

Values are presented as mean ± standard deviation, median (interquartile range) denoted by ^†^, or n (%), as appropriate. Baseline variables were measured during the 365 days before the index date, using the most recent available value before or on the index date for pulmonary function, symptom scores, TCM syndrome score, and laboratory indicators. SLBTD, Shenling Bufei Tongluo Decoction; SMD, standardized mean difference; COPD, chronic obstructive pulmonary disease; FEV_1_, forced expiratory volume in 1 s; FVC, forced vital capacity; GOLD, Global Initiative for Chronic Obstructive Lung Disease; CAT, COPD Assessment Test; mMRC, modified Medical Research Council; TCM, traditional Chinese medicine; ICS, inhaled corticosteroid; LABA, long-acting beta_2_-agonist; LAMA, long-acting muscarinic antagonist.

### 3.4 Missing data, imputation stability, and weighting diagnostics

Missingness of baseline covariates before multiple imputation is summarized in Supplementary Table S1. Missingness was low to moderate across baseline covariates, ranging from 0.0% to 10.4%. The highest missingness was observed for CAT score (10.4%), followed by mMRC dyspnea grade (9.7%), TCM syndrome score (8.5%), and baseline spirometry variables (7.2%). No baseline covariate exceeded the pre-specified 30% missingness threshold. Exposure status, index dates, and the primary post-index clinical event outcome were complete and were not imputed.

Methodological sensitivity analyses for missing-data handling and propensity-weight specification are summarized in Supplementary Table S3. The complete-case analysis was based on 398 patients with fully observed baseline profiles and yielded an estimate consistent with the primary analysis (HR, 0.71; 95% CI, 0.54–0.93; *P* = 0.013). The expanded MICE analysis using 40 imputed datasets (HR, 0.68; 95% CI, 0.53–0.86; *P* = 0.001) and the alternative-seed MICE analysis (HR, 0.69; 95% CI, 0.54–0.88; *P* = 0.003) also produced estimates consistent with the primary analysis, suggesting that the missing-data handling approach did not materially alter the direction or magnitude of the primary association.

The distribution of stabilized propensity score weights and effective sample size is shown in Supplementary Table S2. The mean stabilized weight was approximately 1.00 in both the SLBTD and comparator groups, and the overall sum of stabilized weights was 528.1, close to the original cohort size of 528. Before truncation, the maximum raw stabilized weight was 4.85; after 1st/99th percentile truncation within exposure groups, the maximum stabilized weight was 2.65. The effective sample size increased from 503.2 before truncation to 512.3 after truncation, indicating reduced variance inflation from extreme observations. Analyses using untruncated weights (HR, 0.67; 95% CI, 0.51–0.89; *P* = 0.005) and stricter 5th/95th percentile truncation (HR, 0.69; 95% CI, 0.53–0.91; *P* = 0.007) were consistent with the primary analysis. These diagnostics suggest that the primary findings were not materially sensitive to the imputation setting or the choice of weight truncation threshold.

### 3.5 Time to first moderate-to-severe COPD exacerbation

**Figure 3:**
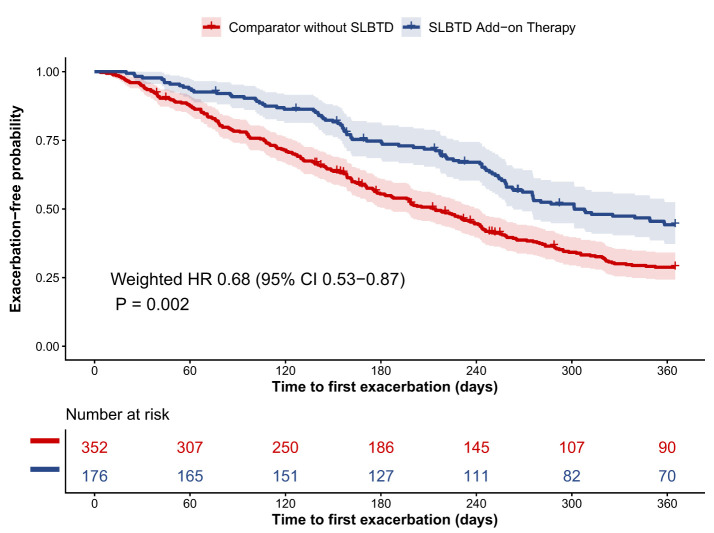
Kaplan–Meier curves for time to first moderate-to-severe COPD exacerbation during the 365-day post-index observation period. SLBTD, Shenling Bufei Tongluo Decoction; COPD, chronic obstructive pulmonary disease.

During the 365-day post-index observation period, the first documented moderate-to-severe COPD exacerbation occurred in 94 of 176 patients (53.4%) in the SLBTD group and 241 of 352 patients (68.5%) in the comparator group. Kaplan–Meier curves showed a higher observed exacerbation-free probability in the SLBTD group throughout the observation period (Figure 3). In the propensity score-weighted Cox proportional hazards model, patients receiving SLBTD add-on therapy had a lower observed risk of first moderate-to-severe COPD exacerbation than patients receiving conventional COPD management without SLBTD (HR, 0.68; 95% CI, 0.53–0.87; *P* = 0.002).

### 3.6 Secondary clinical outcomes

Post-index clinical event outcomes are summarized in Table 4. Consistent with the primary time-to-event analysis, patients receiving SLBTD add-on therapy had fewer recorded exacerbation-related events during the 365-day post-index observation period than comparator patients. The annualized rate of moderate-to-severe COPD exacerbations was 1.34 events per person-year in the SLBTD group and 1.89 events per person-year in the comparator group, corresponding to a weighted rate ratio of 0.71 (95% CI, 0.58–0.86; *P* = 0.001). Severe exacerbation requiring hospitalization occurred in 25 patients (14.2%) in the SLBTD group and 82 patients (23.3%) in the comparator group (OR, 0.55; 95% CI, 0.34–0.89; *P* = 0.014). COPD-related hospitalization, all-cause hospitalization, emergency department visits for COPD, systemic corticosteroid use, and antibiotic use were also recorded less frequently in the SLBTD group than in the comparator group.

**Table 4:** Post-index clinical event outcomes in the retrospective cohort.

Outcome	SLBTD group (*n* = 176)	Comparator group (*n* = 352)	Effect estimate (95% CI)	*P*-value
First moderate-to-severe exacerbation, *n* (%)	94 (53.4)	241 (68.5)	HR 0.68 (0.53–0.87)	0.002
Annualized moderate-to-severe exacerbation rate	1.34	1.89	RR 0.71 (0.58–0.86)	0.001
Any severe exacerbation requiring hospitalization, *n* (%)	25 (14.2)	82 (23.3)	OR 0.55 (0.34–0.89)	0.014
COPD-related hospitalization, *n* (%)	29 (16.5)	89 (25.3)	OR 0.59 (0.37–0.92)	0.021
All-cause hospitalization, *n* (%)	41 (23.3)	116 (33.0)	OR 0.62 (0.41–0.93)	0.020
Emergency department visit for COPD, *n* (%)	36 (20.5)	104 (29.5)	OR 0.62 (0.40–0.96)	0.033
Systemic corticosteroid use for COPD, *n* (%)	62 (35.2)	161 (45.7)	OR 0.64 (0.44–0.93)	0.018
Antibiotic use for COPD, *n* (%)	87 (49.4)	218 (61.9)	OR 0.61 (0.42–0.87)	0.006

Outcomes were ascertained during the 365-day post-index observation period. Hazard ratios were estimated using propensity score-weighted Cox proportional hazards models. Rate ratios were estimated using propensity score-weighted negative binomial regression with observed person-time as an offset. Odds ratios were estimated using propensity score-weighted logistic regression. SLBTD, Shenling Bufei Tongluo Decoction; CI, confidence interval; HR, hazard ratio; RR, rate ratio; OR, odds ratio; COPD, chronic obstructive pulmonary disease.

Changes in symptom scores, pulmonary function indices, and TCM syndrome score are shown in Table 5. Paired CAT measurements were available for 161 patients in the SLBTD group and 312 patients in the comparator group. The CAT score changed by –3.2 ± 4.1 points in the SLBTD group and by –1.1 ± 3.8 points in the comparator group, with an adjusted between-group difference of –2.04 points (95% CI, –3.11 to –0.97; *P* < 0.001). Patients in the SLBTD group also had more favorable changes in mMRC dyspnea grade, TCM syndrome score, FEV_1_% predicted, and FEV_1_/FVC. The adjusted between-group differences were –0.28 for mMRC dyspnea grade (95% CI, –0.45 to –0.11; *P* = 0.001), 2.15 percentage points for FEV_1_% predicted (95% CI, 0.88 to 3.42; *P* = 0.001), 1.22 percentage points for FEV_1_/FVC (95% CI, 0.41 to 2.03; *P* = 0.003), and –3.61 points for TCM syndrome score (95% CI, –4.95 to –2.27; *P* < 0.001).

**Table 5:** Changes in symptom scores, pulmonary function indices, and TCM syndrome score.

Outcome	Paired N (SLBTD/comparator)	SLBTD group (Δ)	Comparator group (Δ)	Adjusted Difference (95% CI), *P* value
CAT score change	161/312	–3.2 ± 4.1	–1.1 ± 3.8	–2.04 (–3.11 to –0.97), *P* < 0.001
mMRC dyspnea grade change	163/314	–0.4 ± 0.6	–0.1 ± 0.5	–0.28 (–0.45 to –0.11), *P* = 0.001
FEV_1_% predicted change	158/305	+0.5 ± 3.9	–1.8 ± 3.5	2.15 (0.88 to 3.42), *P* = 0.001
FEV_1_/FVC change	158/305	+0.8 ± 2.4	–0.5 ± 2.1	1.22 (0.41 to 2.03), *P* = 0.003
TCM syndrome score change	165/318	–5.8 ± 4.5	–2.1 ± 4.1	–3.61 (–4.95 to –2.27), *P* < 0.001

Baseline values were defined as the closest recorded values within 90 days before or on the index date. Post-index values were defined as the recorded values closest to day 365 within the prespecified window. Values for changes are presented as mean ± standard deviation. Between-group differences were estimated using baseline-adjusted propensity score-weighted regression models. For the TCM syndrome score, higher values indicate greater syndrome burden, and negative changes indicate improvement in recorded TCM syndrome burden. SLBTD, Shenling Bufei Tongluo Decoction; CI, confidence interval; CAT, COPD Assessment Test; mMRC, modified Medical Research Council; FEV_1_, forced expiratory volume in 1 s; FVC, forced vital capacity; TCM, traditional Chinese medicine.

### 3.7 Subgroup analyses for the primary outcome

**Figure 4:**
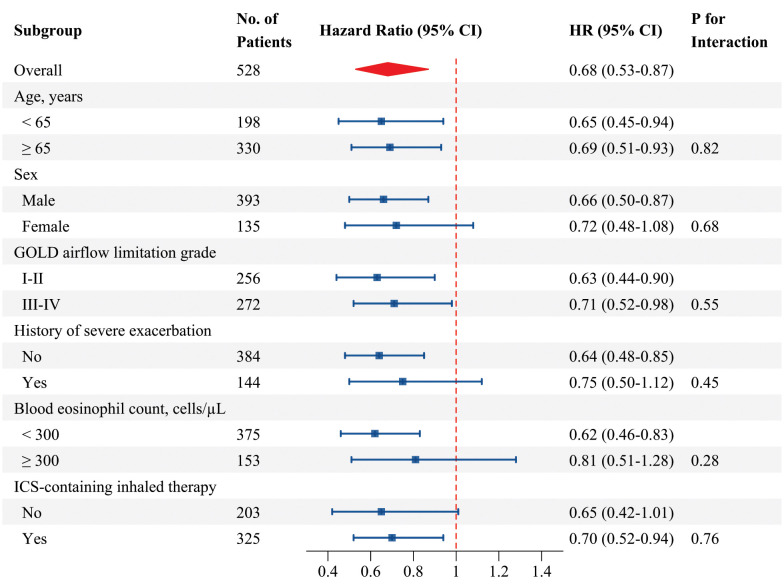
Subgroup forest plot for the primary outcome. Hazard ratios were estimated using propensity score-weighted Cox proportional hazards models with treatment-by-subgroup interaction terms. The primary outcome was first moderate-to-severe COPD exacerbation during the 365-day post-index observation period. SLBTD, Shenling Bufei Tongluo Decoction; COPD, chronic obstructive pulmonary disease; GOLD, Global Initiative for Chronic Obstructive Lung Disease; ICS, inhaled corticosteroid; CI, confidence interval.

Exploratory subgroup analyses for the primary outcome are shown in Figure 4. The association between SLBTD add-on therapy and first moderate-to-severe COPD exacerbation was evaluated across pre-specified subgroups defined by age, sex, GOLD airflow limitation grade, history of severe exacerbation during the baseline window, blood eosinophil count, and ICS-containing inhaled therapy at baseline. Across these subgroups, the hazard ratios ranged from 0.62 to 0.81 and were generally consistent with the overall estimate. All subgroup-specific point estimates favored SLBTD add-on therapy, although confidence intervals crossed unity in several smaller subgroups. No statistically significant interaction was observed for age (*P* for interaction = 0.82), sex (*P* for interaction = 0.68), GOLD airflow limitation grade (*P* for interaction = 0.55), history of severe exacerbation (*P* for interaction = 0.45), blood eosinophil count (*P* for interaction = 0.28), or ICS-containing inhaled therapy (*P* for interaction = 0.76). These findings indicated no evidence of clinically relevant treatment-effect heterogeneity across the pre-specified subgroups.

### 3.8 Sensitivity analyses

Sensitivity analyses for the primary outcome are summarized in Table 6. In the 30-day landmark analysis, the association between SLBTD add-on therapy and a lower risk of first moderate-to-severe COPD exacerbation remained directionally consistent with the main analysis (HR, 0.72; 95% CI, 0.55–0.94; *P* = 0.016). The time-varying exposure analysis produced a similar estimate (HR, 0.70; 95% CI, 0.54–0.91; *P* = 0.008), indicating that the primary finding was not driven by fixed baseline exposure classification. To further evaluate the potential influence of treatment cross-over and late SLBTD initiation among patients initially classified as comparators, we performed a restricted-cohort analysis excluding comparator patients who initiated SLBTD therapy between day 31 and day 365. In this restricted-cohort analysis, the propensity score model and stabilized weights were re-estimated, and the association remained consistent with the main result (HR, 0.66; 95% CI, 0.51–0.85; *P* = 0.001). When the outcome was restricted to severe exacerbations requiring emergency department attendance or hospitalization, SLBTD exposure remained associated with a lower risk of severe exacerbation (HR, 0.61; 95% CI, 0.42–0.89; *P* = 0.010).

**Table 6:** Sensitivity analyses for the primary outcome.

Analysis	Definition	Effect estimate (95% CI)	*P* value
Main analysis	Weighted Cox model for first moderate-to-severe COPD exacerbation	HR 0.68 (0.53–0.87)	0.002
30-day landmark analysis	Exposure defined during days 0–30; outcome follow-up from day 31	HR 0.72 (0.55–0.94)	0.016
Time-varying exposure analysis	SLBTD modeled as a time-varying covariate	HR 0.70 (0.54–0.91)	0.008
Restricted-cohort analysis	Excluding late SLBTD initiators in the comparator group during follow-up	HR 0.66 (0.51–0.85)	0.001
Severe-exacerbation-only analysis	First COPD exacerbation requiring emergency department attendance or hospitalization	HR 0.61 (0.42–0.89)	0.010

The main analysis used a propensity score-weighted Cox proportional hazards model. The 30-day landmark analysis excluded patients with a moderate-to-severe COPD exacerbation, death, or no available record before day 30 and started outcome follow-up on day 31. The time-varying exposure analysis classified person-time according to the timing of the first recorded SLBTD prescription. The restricted-cohort analysis excluded comparator patients who initiated SLBTD therapy between day 31 and day 365, re-estimated the propensity score model and stabilized weights in the restricted cohort, and refitted the weighted Cox model for the primary outcome. The severe-exacerbation-only analysis counted only exacerbations requiring emergency department attendance or hospitalization. SLBTD, Shenling Bufei Tongluo Decoction; COPD, chronic obstructive pulmonary disease; CI, confidence interval; HR, hazard ratio.

### 3.9 Safety outcomes

Recorded safety outcomes during the 365-day post-index observation period are presented in Table 7. Any recorded adverse event occurred in 14 patients (8.0%) in the SLBTD group and 31 patients (8.8%) in the comparator group, with no evidence of an increased risk associated with SLBTD add-on therapy (OR, 0.89; 95% CI, 0.46–1.72; *P* = 0.724). Gastrointestinal symptoms were documented in 6 patients (3.4%) in the SLBTD group and 11 patients (3.1%) in the comparator group (OR, 1.09; 95% CI, 0.40–2.98; *P* = 0.865). Allergic reactions, dizziness, palpitations, liver enzyme elevations, renal function abnormalities, and blood urea nitrogen abnormalities were infrequent in both groups, and none showed a statistically significant between-group difference. Treatment discontinuation recorded in SLBTD prescription records occurred in three patients (1.7%) in the SLBTD group. All-cause death documented in hospital records occurred in 4 patients (2.3%) in the SLBTD group and 11 patients (3.1%) in the comparator group (OR, 0.72; 95% CI, 0.23–2.28; *P* = 0.578).

**Table 7:** Recorded safety outcomes during the post-index observation period.

Safety outcome	SLBTD group (*n* = 176)	Comparator group (*n* = 352)	Effect estimate (95% CI)	*P*-value
Any recorded adverse event, *n* (%)	14 (8.0)	31 (8.8)	OR 0.89 (0.46–1.72)	0.724
Gastrointestinal symptoms, *n* (%)	6 (3.4)	11 (3.1)	OR 1.09 (0.40–2.98)	0.865
Allergic reaction, *n* (%)	2 (1.1)	5 (1.4)	OR 0.79 (0.15–4.15)	0.785
Dizziness, *n* (%)	3 (1.7)	7 (2.0)	OR 0.85 (0.22–3.32)	0.817
Palpitations, *n* (%)	1 (0.6)	3 (0.9)	OR 0.66 (0.07–6.42)	0.722
ALT or AST >3 × upper limit of normal, *n* (%)	2 (1.1)	5 (1.4)	OR 0.79 (0.15–4.15)	0.784
Serum creatinine increase ≥50% from baseline, *n* (%)	1 (0.6)	3 (0.9)	OR 0.66 (0.07–6.38)	0.722
Blood urea nitrogen above upper reference limit, *n* (%)	4 (2.3)	9 (2.6)	OR 0.88 (0.27–2.89)	0.835
Treatment discontinuation recorded in prescription records, *n* (%)	3 (1.7)	–	–	–
All-cause death documented in hospital records, *n* (%)	4 (2.3)	11 (3.1)	OR 0.72 (0.23–2.28)	0.578

Safety outcomes were identified from outpatient records, inpatient records, laboratory information systems, and prescription records during the 365-day post-index observation period. Observed counts and percentages are unweighted. Odds ratios were estimated using propensity score-weighted logistic regression with robust standard errors where model estimation was feasible. Safety outcome categories were not mutually exclusive. Treatment discontinuation was evaluated only among patients with recorded SLBTD prescriptions and was therefore not compared between groups. SLBTD, Shenling Bufei Tongluo Decoction; CI, confidence interval; OR, odds ratio; ALT, alanine aminotransferase; AST, aspartate aminotransferase.

## 4 Discussion

### 4.1 Principal findings

In this single-center retrospective real-world cohort of 528 patients with stable COPD and a frequent-exacerbator phenotype, patients receiving SLBTD add-on therapy had a lower observed risk of first moderate-to-severe COPD exacerbation during the 365-day post-index observation period than patients receiving conventional management alone. This pattern remained directionally consistent across secondary event outcomes, including annualized exacerbation rate, severe exacerbation requiring hospitalization, COPD-related hospitalization, all-cause hospitalization, emergency department visits for COPD, systemic corticosteroid use, and antibiotic use. Patients receiving SLBTD also showed more favorable changes in CAT score, mMRC dyspnea grade, TCM syndrome score, and pulmonary function indices. No increased risk of recorded adverse events, hepatic laboratory abnormalities, renal laboratory abnormalities, or all-cause death was observed. Although the main analysis classified comparator patients partly by the absence of SLBTD prescription within the first 30 days after index, the 30-day landmark analysis, time-varying exposure analysis, and restricted-cohort analysis yielded estimates consistent with the primary result, suggesting that the observed association was not primarily driven by this exposure-classification strategy.

These findings are clinically relevant because exacerbations are central events in COPD progression. Exacerbation-prone patients have a reproducible tendency toward future exacerbations, and frequent exacerbations have been linked to accelerated lung function decline and worse long-term outcomes (1, 5). Recent evidence also confirms that COPD exacerbation-related hospitalization is followed by substantial mortality and readmission risk, and that exacerbation history remains a key element for estimating future COPD outcomes (3, 6). Therefore, the present results should be interpreted as observational evidence supporting further evaluation of adjunctive strategies for stable COPD patients with a frequent-exacerbator phenotype.

### 4.2 Clinical interpretation and potential mechanisms

The observed association between SLBTD use and lower recorded exacerbation-related event rates should be interpreted as adjunctive real-world evidence rather than proof of causal efficacy. Contemporary COPD management emphasizes symptom reduction and exacerbation prevention, and randomized trials of inhaled triple therapy and targeted biologic therapy have shown that treatment intensification can reduce moderate-to-severe exacerbations in selected high-risk COPD populations (2, 22–24). However, high-risk patients may continue to experience symptoms and recurrent exacerbations despite conventional therapy, leaving room for add-on strategies that can be evaluated prospectively and in routine-care settings. The direction of our findings is consistent with prior clinical studies of Chinese herbal medicine as adjunctive therapy in stable COPD. Previous studies of YuPingFeng granules, JianPiYiFei II granules, Bu-Fei Yi-Shen granules, and TCM pattern-based therapy combined with conventional Western medicine have reported reductions in acute exacerbations, exacerbation-related hospitalization, or symptom burden in selected COPD populations (8–11). Mechanistically, COPD exacerbations are heterogeneous events involving airway inflammation, host defense, infection, mucus dysfunction, and systemic vulnerability (25, 26). Experimental work on related Bufei formulas suggests potential immunomodulatory effects, including regulation of Th17/Treg balance (27). These mechanisms remain hypothesis-generating in the present study and require prospective biological validation.

### 4.3 Strengths, limitations, and implications

This study has several strengths. The cohort was restricted to stable COPD patients with a frequent-exacerbator phenotype, which created a clinically meaningful high-risk population. The design defined a clear index date, a 365-day baseline assessment window, and a 365-day post-index outcome ascertainment window. Exposure was defined from electronic prescription records, and stabilized propensity score weighting achieved good measured covariate balance. The outcome assessment covered exacerbation-related events, medication use, symptom scores, pulmonary function indices, TCM syndrome score, and recorded safety outcomes.

Several limitations must be acknowledged. First, this was a single-center retrospective study, and treatment was not randomized; residual confounding by unmeasured or incompletely measured factors cannot be excluded. Although the propensity score model included multiple core prognostic variables and treatment-related covariates, including smoking status, COPD severity, previous exacerbation history, pulmonary function, symptom burden, comorbidities, baseline COPD management, long-term oxygen therapy, and calendar year of the index date, several important factors could not be reliably incorporated into the primary model. These included smoking intensity, influenza and pneumococcal vaccination history, socioeconomic position, and adherence to home oxygen therapy. These factors may influence COPD prognosis, healthcare-seeking behavior, access to add-on treatment, medication adherence, and the likelihood of documented exacerbation-related encounters. Therefore, residual confounding related to these variables may have affected the estimated association between SLBTD add-on therapy and clinical outcomes. The study period also overlapped with the COVID-19 pandemic. Calendar year of the index date was included to partially account for period effects, but COVID-19-related factors could not be comprehensively captured from retrospective hospital records. In particular, COVID-19 infections diagnosed outside the hospital, mild infections not leading to medical encounters, changes in healthcare access, and pandemic-related changes in respiratory infection patterns may have influenced both COPD exacerbation risk and outcome ascertainment. Therefore, unmeasured or incompletely recorded COVID-19-related factors should be considered when interpreting the findings. Second, SLBTD exposure was based on electronic prescription records, which may not fully capture actual intake, decoction quality outside the hospital system, or day-to-day adherence. Although we supplemented the exposure description by reporting the recorded treatment duration and treatment-course distribution and reviewed hospital procedure records for concomitant non-oral TCM-related interventions, undocumented TCM interventions received outside the hospital system could not be fully captured. Therefore, residual confounding related to actual SLBTD adherence, out-of-hospital TCM use, or unrecorded non-oral TCM interventions cannot be completely excluded. In addition, although the TCM syndrome score was recorded using a standardized hospital assessment form and showed good internal consistency and inter-rater reliability in the retrospective reliability audit, it includes clinician-rated symptom and sign components. Therefore, it may be more subjective than spirometric, hospitalization, or medication-use outcomes, and residual measurement bias in TCM outcome evaluation cannot be fully excluded. The generalizability of the findings is also limited by the single-center setting and the regional nature of the cohort. All patients were treated at one hospital in Liaoning Province, and local referral patterns, climate, healthcare access, COPD management practices, and TCM use patterns may differ from those in other regions of China or other countries. In addition, SLBTD in this study referred specifically to the hospital's fixed 12-component in-house decoction formula recorded in the electronic prescription system. The findings should therefore not be directly generalized to commercially available TCM products, modified versions of SLBTD, other Bufei Tongluo-related formulas, or formulations prepared under different prescription, processing, decoction, or quality-control conditions. Multicenter prospective studies across different geographic regions, with standardized formulation preparation and quality monitoring, are needed before broader generalization can be made. Another consideration is that the latest GOLD 2026 report lowered the exacerbation-history threshold for considering treatment escalation by recognizing the prognostic importance of even one moderate exacerbation. The present study intentionally retained the more stringent and historically established frequent-exacerbator definition of two or more moderate exacerbations or at least one severe exacerbation in the previous year to focus on a higher-risk and more clinically homogeneous population. Therefore, the findings should not be extrapolated to COPD patients with only one prior moderate exacerbation, who may meet the updated GOLD treatment-escalation threshold but were not represented in this cohort. Safety outcomes should also be interpreted cautiously because they were obtained through retrospective passive monitoring of hospital and outpatient records rather than active prospective adverse-event surveillance. Mild, transient, self-limited, or out-of-hospital adverse events may not have been reported by patients or recorded in the medical records. Therefore, the possibility of underreporting mild adverse events cannot be ruled out, and the safety findings should be interpreted as recorded safety outcomes rather than definitive evidence of the absence of adverse effects.

Overall, this study provides real-world evidence that patients receiving SLBTD add-on therapy had a lower recorded exacerbation burden and more favorable symptom-related outcomes than patients receiving conventional management alone, without an observed increase in recorded safety events. Given the observational design, these findings should be interpreted as hypothesis-generating and support further multicenter prospective studies and, ideally, randomized controlled trials with standardized exposure monitoring, active safety surveillance, and mechanistic biomarker assessment.

## 5 Conclusion

In this retrospective real-world cohort study of stable COPD patients with a frequent-exacerbator phenotype, patients receiving Shenling Bufei Tongluo Decoction as add-on therapy to conventional COPD management had a lower observed risk of first moderate-to-severe COPD exacerbation during the 365-day post-index observation period than patients receiving conventional management alone. Patients receiving SLBTD add-on therapy also had a lower annualized exacerbation rate, fewer recorded exacerbation-related hospitalizations and emergency department visits, lower recorded systemic corticosteroid and antibiotic use, and more favorable changes in CAT score, mMRC dyspnea grade, pulmonary function indices, and TCM syndrome score. No increased risk of recorded adverse events, hepatic laboratory abnormalities, renal laboratory abnormalities, or all-cause death was observed among patients receiving SLBTD add-on therapy. These findings provide observational real-world evidence that may support further evaluation of SLBTD as an adjunctive option for high-risk stable COPD patients with recurrent exacerbation history.

## Data Availability

The original contributions presented in the study are included in the article/Supplementary material, further inquiries can be directed to the corresponding author.
