# Supplementary material for: Clinical outcomes associated with Shenling Bufei Tongluo Decoction as add-on therapy in stable COPD patients with a frequent-exacerbator phenotype: a retrospective real-world cohort study
Source: Front Med (Lausanne). 2026 Jul 8;13:1865770. doi: 10.3389/fmed.2026.1865770 (PMC13388256; doi:10.3389/fmed.2026.1865770)
Supplement: Supplementary file 1 [file Data_Sheet_1.PDF]

Completed STROBE Checklist for Cohort Studies

Manuscript title: Clinical Outcomes Associated With Shenling Bufei Tongluo Decoction as Add-on Therapy in Stable COPD Patients With a Frequent-Exacerbator Phenotype: A Retrospective Real-World Cohort Study

Checklist used: STROBE Statement—Checklist of items that should be included in reports of cohort studies.

Section / Topic	Item No.	Recommendation	Reported in manuscript / location
Title and abstract	1(a)	Indicate the study's design with a commonly used term in the title or the abstract.	Title: includes "Retrospective Real-World Cohort Study".<br>Abstract, Methods: states that this was a retrospective real-world cohort study evaluating SLBTD add-on therapy in stable COPD patients with a frequent-exacerbator phenotype.
Title and abstract	1(b)	Provide in the abstract an informative and balanced summary of what was done and what was found.	Abstract, Background/Methods/Results/Conclusion: summarizes rationale, cohort design, exposure and comparator, propensity score weighting, primary and secondary outcomes, key effect estimates, symptom and pulmonary-function outcomes, and recorded safety outcomes. The revised abstract uses observational language and states that findings should be interpreted as associations rather than causal efficacy.
Introduction<br>Background/rationale	2	Explain the scientific background and rationale for the investigation being reported.	Introduction: describes COPD exacerbations as clinically important events, the frequent-exacerbator phenotype as a high-risk subgroup, the routine use of Chinese herbal medicine in COPD care in China, and the limited real-world evidence for SLBTD in this population.
Introduction<br>Objectives	3	State specific objectives, including any prespecified hypotheses.	End of Introduction: states that the study aimed to evaluate clinical outcomes associated with SLBTD add-on therapy among stable COPD patients with a frequent-exacerbator phenotype in routine clinical practice. The primary and secondary outcomes are listed.
Methods<br>Study design	4	Present key elements of study design early in the paper.	Section 2.1, Study design, setting, and ethics: identifies the study as a single-center retrospective real-world cohort study using routine clinical records; clarifies that treatment was not assigned by investigators and that exposure was defined retrospectively from electronic prescription records.
Methods<br>Setting	5	Describe the setting, locations, and relevant dates, including periods of recruitment, exposure, follow-up, and data collection.	Section 2.1 and Section 2.2: reports the Affiliated Hospital of Liaoning University of Traditional Chinese Medicine as the study setting; data extraction period was 1 January 2019 to 31 December 2025; eligible index dates were 1 January 2020 to 31 December 2024; each patient had a 365-day baseline assessment window and a 365-day post-index outcome ascertainment window.
Methods<br>Participants	6(a)	Give the eligibility criteria, and the sources and methods of selection of participants. Describe methods of follow-up.	Section 2.2, Data source and cohort identification: lists data sources and de-duplication method. Inclusion criteria include age >=40 years, physician-diagnosed and spirometry-confirmed COPD, stable COPD, frequent-exacerbator phenotype, and available records. Exclusion criteria include acute exacerbation at index, asthma as primary respiratory diagnosis, lung cancer, active tuberculosis, interstitial lung disease, missing exposure/index/primary outcome information, and concurrent COPD-targeted oral Chinese herbal formulas. Follow-up/outcome ascertainment was based on outpatient records, inpatient records, prescription records, pulmonary-function reports, laboratory systems, and structured follow-up records during the 365-day post-index period.
Methods<br>Participants	6(b)	For matched studies, give matching criteria and number of exposed and unexposed.	Not applicable. This was not a matched cohort study. The final cohort included 176 patients receiving SLBTD add-on therapy and 352 comparator patients receiving conventional COPD management without SLBTD. Propensity score weighting, rather than matching, was used to reduce measured baseline imbalance.
Methods<br>Variables	7	Clearly define all outcomes, exposures, predictors, potential confounders, and effect modifiers. Give diagnostic criteria, if applicable.	Sections 2.3-2.8: defines stable COPD, moderate and severe acute exacerbations, frequent-exacerbator phenotype, SLBTD exposure, comparator definition, index date, baseline and post-index windows, primary and secondary outcomes, safety outcomes, baseline covariates, and prespecified subgroup variables/effect modifiers. COPD diagnosis was based on post-bronchodilator FEV1/FVC <0.70.
Methods<br>Data sources/measurement	8	For each variable of interest, give sources of data and details of methods of assessment (measurement). Describe comparability of assessment methods if there is more than one group.	Sections 2.2, 2.4, 2.5, and Tables 1-2: data came from electronic medical records, outpatient and inpatient records, electronic prescriptions, pulmonary-function reports, laboratory information systems, and structured follow-up records. Exposure was identified from electronic prescription records. Outcomes were ascertained using the same hospital data sources in both exposure groups. The revised manuscript describes SLBTD prescription characteristics, treatment duration, concomitant TCM-related interventions, measurement windows for symptom and pulmonary-function outcomes, and retrospective passive monitoring of safety outcomes.
Methods	9	Describe any efforts to address potential sources of bias.	Sections 2.6-2.8, Section 3.4, and Discussion: propensity score weighting was used to reduce measured

Section / Topic	Item No.	Recommendation	Reported in manuscript / location
Bias			baseline imbalance; stabilized weights were truncated to limit extreme weights; covariate balance was assessed using standardized mean differences and a Love plot; multiple imputation addressed missing baseline covariates; missing-data and weight diagnostics were reported in Section 3.4 and Supplementary Tables S1-S3; sensitivity analyses included 30-day landmark, time-varying exposure, restricted-cohort, and severe-exacerbation-only analyses. The revised Discussion explicitly addresses residual confounding, selection bias, TCM-score subjectivity, passive safety monitoring, and limited generalizability.
Methods<br>Study size	10	Explain how the study size was arrived at.	Section 2.8, Statistical analysis: states that no formal sample-size calculation was performed because the study included all eligible patients within the predefined index period.
Methods<br>Quantitative variables	11	Explain how quantitative variables were handled in the analyses. If applicable, describe which groupings were chosen and why.	Sections 2.6-2.8: continuous variables were summarized as mean +/- SD or median (IQR), and categorical variables as n (%). Baseline values used the most recent valid value before or on the index date. Change-score analyses used paired baseline and post-index measurements. Prespecified subgroup groupings included age <65 vs >=65 years, GOLD I-II vs III-IV, and blood eosinophil count <300 vs >=300 cells/microL.
Methods<br>Statistical methods	12(a)	Describe all statistical methods, including those used to control for confounding.	Sections 2.7-2.8: describes propensity score estimation by multivariable logistic regression, stabilized inverse probability of treatment weighting, weight truncation at the 1st/99th percentiles, covariate balance assessment using standardized mean differences, weighted Cox models with robust standard errors for the primary outcome, weighted negative binomial regression for exacerbation counts, weighted logistic regression for binary outcomes, and baseline-adjusted weighted regression for continuous change outcomes.
Methods<br>Statistical methods	12(b)	Describe any methods used to examine subgroups and interactions.	Section 2.8: prespecified subgroup analyses by age, sex, GOLD airflow limitation grade, severe exacerbation history, blood eosinophil count, and ICS-containing inhaled therapy; weighted Cox models with treatment-by-subgroup interaction terms were used. Results are reported in Section 3.7 and Figure 4.
Methods<br>Statistical methods	12(c)	Explain how missing data were addressed.	Section 2.6: patients with missing exposure status, index date, or primary outcome information were excluded. Missing baseline covariates were handled using multiple imputation by chained equations (MICE) with 20 imputed datasets; the imputation model included exposure status, primary outcome status, time to event or censoring, baseline covariates, and major secondary event outcomes. Variables with >30% missingness were excluded from primary propensity score and outcome models. Missingness and imputation-stability diagnostics are reported in Section 3.4 and Supplementary Tables S1 and S3.
Methods<br>Statistical methods	12(d)	If applicable, explain how loss to follow-up was addressed.	Section 2.8: patients were censored at first moderate-to-severe exacerbation, death, last available clinical record, or day 365. Follow-up was based on routine hospital records over the fixed 365-day post-index observation window.
Methods<br>Statistical methods	12(e)	Describe any sensitivity analyses.	Section 2.8 and Section 3.8: sensitivity analyses included 30-day landmark analysis, time-varying exposure analysis, restricted-cohort analysis excluding late SLBTD initiators in the comparator group, and severe-exacerbation-only analysis. Additional missing-data and weight-truncation sensitivity analyses are reported in Section 3.4 and Supplementary Tables S2-S3.
Results<br>Participants	13(a)	Report numbers of individuals at each stage of study--eg numbers potentially eligible, examined for eligibility, confirmed eligible, included in the study, completing follow-up, and analysed.	Section 3.1 and Figure 1: reports 2,436 COPD patients identified, 1,568 with spirometry-confirmed COPD, 982 eligible for phenotype assessment, 612 meeting frequent-exacerbator phenotype, 84 excluded, and 528 included in the final cohort.
Results<br>Participants	13(b)	Give reasons for non-participation at each stage.	Section 3.1 and Figure 1: reasons for exclusion are reported, including acute exacerbation at index, asthma as primary respiratory diagnosis, lung cancer/active tuberculosis/interstitial lung disease, missing exposure or primary outcome information, and unclear exposure classification due to concurrent COPD-targeted oral Chinese herbal formulas.
Results<br>Participants	13(c)	Consider use of a flow diagram.	Figure 1: flow diagram of retrospective cohort identification and exposure classification.
Results<br>Descriptive data	14(a)	Give characteristics of study participants (eg demographic, clinical, social) and information on exposures and potential confounders.	Section 3.3 and Table 3: baseline demographic, clinical, treatment, comorbidity, pulmonary-function, symptom, TCM syndrome, and laboratory characteristics are reported separately for the SLBTD and comparator groups, including unweighted and weighted standardized mean differences. Section 3.2 and Table 2 report SLBTD treatment duration and concomitant TCM-related interventions.
Results<br>Descriptive data	14(b)	Indicate number of participants with missing data for each variable of interest.	Section 2.6 and Section 3.4 describe missing-data handling and diagnostics. Supplementary Table S1 reports the number and percentage of missing values for each baseline covariate and the corresponding imputation approach.

Section / Topic	Item No.	Recommendation	Reported in manuscript / location
Results<br>Descriptive data	14(c)	Summarise follow-up time (eg, average and total amount).	Sections 2.1, 2.4, and 2.8: each patient had a predefined 365-day post-index outcome ascertainment window, with censoring at first event, death, last available clinical record, or day 365. Table 4 reports annualized exacerbation rates, and statistical models used observed person-time as an offset for count outcomes.
Results<br>Outcome data	15	Report numbers of outcome events or summary measures over time.	Sections 3.5, 3.6, and 3.9 and Tables 4-5 and 7: reports primary exacerbation events, annualized exacerbation rate, severe exacerbation, hospitalization, emergency department visit, systemic corticosteroid and antibiotic use, continuous outcome changes, and recorded safety outcomes separately by exposure group.
Results<br>Main results	16(a)	Give unadjusted estimates and, if applicable, confounder-adjusted estimates and their precision. Make clear which confounders were adjusted for and why they were included.	Tables 3-7 and Sections 2.6-2.8/3.5-3.9: observed counts/percentages or means are shown by group, and propensity score-weighted effect estimates with 95% CIs and P values are reported for the primary, secondary, subgroup, sensitivity, and safety analyses. The propensity score model covariates and rationale are described in Sections 2.6 and 2.7.
Results<br>Main results	16(b)	Report category boundaries when continuous variables were categorized.	Sections 2.8 and 3.7/Figure 4: subgroup category boundaries are reported for age (<65 vs >=65 years), GOLD airflow limitation grade (I-II vs III-IV), and blood eosinophil count (<300 vs >=300 cells/microL). Table 2 reports SLBTD treatment-duration categories (<30, 30-59, 60-89, and >=90 days).
Results<br>Main results	16(c)	If relevant, consider translating estimates of relative risk into absolute risk for a meaningful time period.	Sections 3.5-3.6 and Table 4: provides absolute event counts and percentages during the 365-day post-index observation period, together with annualized exacerbation rates, alongside HR/RR/OR estimates.
Results<br>Other analyses	17	Report other analyses done--eg analyses of subgroups and interactions, and sensitivity analyses.	Section 3.7 and Figure 4: exploratory subgroup analyses and interaction P values. Section 3.8 and Table 6: sensitivity analyses for the primary outcome, including 30-day landmark, time-varying exposure, restricted-cohort, and severe-exacerbation-only analyses. Additional missing-data and weight-truncation sensitivity analyses are reported in Section 3.4 and Supplementary Tables S2-S3.
Discussion<br>Key results	18	Summarise key results with reference to study objectives.	Section 4.1, Principal findings: summarizes the primary and secondary observed associations, symptom and pulmonary-function changes, safety outcomes, and consistency of sensitivity analyses in relation to the study objective.
Discussion<br>Limitations	19	Discuss limitations of the study, taking into account sources of potential bias or imprecision. Discuss both direction and magnitude of any potential bias.	Section 4.3: discusses single-center retrospective design, non-randomized treatment, residual confounding from unmeasured or incompletely measured factors, COVID-19-related factors, exposure/adherence limitations, out-of-hospital TCM use, TCM syndrome-score subjectivity, limited generalizability, and passive safety monitoring with possible underreporting of mild adverse events.
Discussion<br>Interpretation	20	Give a cautious overall interpretation of results considering objectives, limitations, multiplicity of analyses, results from similar studies, and other relevant evidence.	Sections 4.1-4.3 and Section 5, Conclusion: revised language consistently describes results as observational associations rather than causal efficacy; discusses consistency with prior studies and the need for prospective multicenter studies and randomized controlled trials.
Discussion<br>Generalisability	21	Discuss the generalisability (external validity) of the study results.	Section 4.3: explicitly states that findings are limited by the single-center Liaoning cohort and the hospital's fixed 12-component in-house SLBTD decoction formula; findings should not be directly generalized to commercial TCM products, modified formulas, other Bufei Tongluo-related formulas, or other geographic/healthcare settings without further evaluation.
